# The Use of Neuroscience and Psychological Measurement in England's Court of Protection

**DOI:** 10.3389/fpsyt.2020.570709

**Published:** 2020-12-07

**Authors:** Andrew McWilliams, Stephen M. Fleming, Anthony S. David, Gareth Owen

**Affiliations:** ^1^Mental Health Ethics and Law Research Group, Department of Psychological Medicine, Institute of Psychiatry, Psychology and Neuroscience, King's College London, London, United Kingdom; ^2^Metacognition Group, Wellcome Centre for Human Neuroimaging, University College London, London, United Kingdom; ^3^Department of Experimental Psychology, University College London, London, United Kingdom; ^4^Institute of Mental Health, University College London, London, United Kingdom

**Keywords:** court of protection, psychometric, neuroscience, neurolaw, neuroethics, minimally conscious, capacity, mental capacity

## Abstract

The 2005 Mental Capacity Act of England and Wales provides a description in statute law of a test determining if a person lacks “mental capacity” to take a particular decision and describes how the “best interests” of such a person should be determined. The Act established a new Court of Protection (CoP) to hear cases related to the Act and to rule on disputes over mental capacity. The court gathers a range of evidence, including reports from clinicians and experts. Human rights organisations and others have raised concerns about the nature of assessments for incapacity, including the role of brain investigations and psychometric tests.

**Aim:** Describe use and interpretation of structured measures of psychological and brain function in CoP cases, to facilitate standardisation and improvement of practices, both in the courtroom and in non-legal settings.

**Method:** Quantitative review of case law using all CoP judgments published until 2019. The judgments (*n* = 408) were read to generate a subset referring to structured testing (*n* = 50). These were then examined in detail to extract the nature of the measurements, circumstances of their use and features of interpretation by the court.

**Results:** The 408 judgments contained 146 references to structured measurement of psychological or brain function, spread over 50 cases. 120/146 (82.2%) referred to “impairment of mind or brain,” with this being part of assessment for incapacity in 58/146 (39.7%). Measurement referred on 25/146 (17.1%) occasions to “functional decision-making abilities.” Structured measures were used most commonly by psychiatrists and psychologists. Psychological measurements comprised 66.4% of measures. Neuroimaging and electrophysiology were presented for diagnostic purposes only. A small number of behavioural measures were used for people with disorders of consciousness. When assessing incapacity, IQ and the Mini-Mental-State Examination were the commonest measures. A standardised measure of mental capacity itself was employed just once. Judges rarely integrated measurements in their capacity determinations.

**Conclusion:** Structured testing of brain and psychological function is used in limited ways in the Court of Protection. Whilst there are challenges in creating measures of capacity, we highlight an opportunity for the neuroscience community to improve objectivity in assessment, inside and outside the courtroom.

## Introduction

The Court of Protection (CoP) of England and Wales was established in its current form as a specialised court dealing with matters arising from the Mental Capacity Act 2005 (MCA 2005)^1^. Judges in the CoP hear cases discussing fundamental issues of human experience, including personal autonomy and the desire for self-determination in matters of life and death. For example, the court regularly hears cases concerning a person choosing whether or not to live independently of support, to make gifts of large sums of money or to refuse an offer of life-saving surgery. The MCA 2005 provides a description in statute law of a test to determine whether a person lacks the “mental capacity” to take a particular decision and goes on to describe what must be done for a person who lacks that capacity in their best interests. The CoP therefore hears cases where there are disputes over outcomes of capacity or best interests assessment, where guidance is sought about those matters, or to rule on points of law resulting from the MCA 2005 itself.

The Act is distinctive for several reasons, not least in that it begins with five overarching principles: (1) there is a presumption that any person (referred to as “P” in the court) has capacity, so the job of an assessor is to establish if this presumption should be displaced; (2) all practicable steps must be taken to help P make a decision for themselves; (3) people who have mental capacity may make choices which others see as unwise; (4) decisions taken on behalf of people who lack capacity must be taken in their best interests; and (5) decisions must only be taken on behalf of people if there is no less restrictive way of achieving the desired aim. Capacity is not globally present or absent for any P, but is tied to the moment in time at which a decision is being taken and to the content of that decision, such as a specific choice about healthcare, finances or residence.

The criteria for assessing incapacity are delineated in the MCA 2005 in two interlinked sections. First (MCA 2005 section 2), sets out that incapacity means to be unable to make a decision for oneself in relation to the matter “because of an impairment of, or a disturbance in the functioning of, the mind or brain.” Second (MCA 2005 section 3), sets out what to be unable means in terms of functional inabilities: understanding the relevant information; retaining that information; using or weighing that information to reach a decision; communicating a choice. Any one of these functional inabilities, if arising “because of” an impairment of mind or brain is incapacity.

The judge is the arbiter of all determinations in court and the evidence considered by the judge can encompass many forms. Although some evidence uses reports and interviews undertaken especially for court proceedings by social and healthcare professionals, much other evidence includes information from other sources, including from routine care records. This means that as neuroscience develops—for use in clinical or social settings—measurements that have some face validity for decision-making, these neuroscientific measures will increasingly appear in court. The relevance and validity of their use for legal purposes will thus need to be considered. The developing field of neurolaw seeks to provide a neuroscientific perspective on legal questions, through use of neuropsychology, neuroimaging, and other scientific methods. However, neuroscience faces challenges resulting from the sheer complexity of the subject matters in question, with biology, psychology and neuroscience intersecting across philosophy and disability law to speak about questions of vital societal importance. For instance, models of mental capacity must encompass individual factors involved in making a decision, whilst allowing choices which an observer may see as not “reasonable” but which are still a capacitous expression of P's wishes (1).

One challenge for neuroscientists is assessing the relevance of damage to the brain or mental disorder to decision-making capacity in general. Another, related to this, is the so-called “G2i” (Group to Individual) problem (2). This states that research is able to provide population norms for biological parameters typically seen in people who have a certain behavioural characteristic (e.g., impaired capacity). Yet demonstrating that an individual has a parameter typical of a person with a certain characteristic is not the same as showing that they in fact possess that characteristic. Men might in general behave more aggressively than women, but this does not entail that all humans with XY chromosomes are aggressive. While a neuroscientific measurement might show a particular P to have a decision-making process or brain which displays certain parameters commonly found with people who lack capacity, the court will not regard this as determinative of impairment or decision-making incapacity in that particular P.

Nonetheless, a recent systematic review of legal publications during 2016 (3) concluded that there was some increasing appetite for biological psychiatric models to explain human behaviour and mental states. A UK study of 1585 cases heard in the Court of Appeal (Criminal Division) found neuroscientific evidence was used in ~1% of cases, with clear indications that the court had used the neuroscience to reach their conclusions (4). However, there are concerns that the power of neuroscience to address questions in court has been over-stated. Scientific experts may be commenting on questions which science cannot in fact yet answer, or which are not relevant to the legal questions the court wishes to know about an individual (5). The neuroscientific view is based on an essentially biological model of normative physiology and brain function as being necessary (or even sufficient) for decision-making—an assumption which is challenged by disability rights and other groups (6). Additionally, the court may lack the technical knowledge to interpret or use the science it hears (7).

The same measurement tools which scientists develop for research purposes, such as understanding how systems in the brain determine cognition, motivation and behaviour, are often used in related forms in clinical settings, and vice-versa. For instance, classical clinical neuropsychological tests are often used in modified form by cognitive neuroscientists seeking to delineate the brain basis of particular psychological abilities, such as memory or decision-making. Hence there may be instances in which the tools of laboratory psychology and cognitive neuroscience can be used to inform on issues of either (1) impairments of brain and mind or (2) decision-making function. In turn, this suggests that evidence from neuroscience and psychology may be useful when assessing P to prepare professional evidence to be heard in court. However, the extent to which this is the case in the CoP remains unknown.

Undertaking empirical research into practices in the CoP is not straight forward, due to the limited ways to access the wide variety of relevant data about cases and hearings. Evidence heard in court may come from a variety of sources. MCA 2005 s.49 grants the CoP power to order oral or written reports from NHS health bodies and local authorities connected to P. Documents made originally for non-legal purposes, such as routine healthcare records, may also be produced as evidence. Reports can be requested from court-appointed medical or other experts or Special Visitors, who may meet P only once. Researchers have gained access to paper and electronic court files, but this has been possible only with extensive co-operation of CoP staff spread throughout the jurisdiction (8).

Following concerns raised about the transparency of processes in the court system, a CoP practice directive set a precedent to encourage publication of more judgments (9). The drivers of how judges choose whether to request publication of a particular judgment are complex, but this formal reporting of a small proportion judgments gives a window into practices of the CoP (10). The *British and Irish Legal Information Institute* (BAILII) is an online, publicly accessible legal database, which contains these judgments near-comprehensively.

We therefore were able to perform a retrospective quantitative review of how structured measures from psychology and neuroscience are presented and used in the CoP in order to: (a) provide a map of current practice, (b) allow informed critique of current practice, and (c) guide recommendations for improving measurement and measurement practice.

## Materials and Methods

Cases are labelled by the court system with an identifier referring to the type of court which heard the case, with “EWCOP” being the identifier attached to cases heard in the current day CoP. Staff at BAILII supplied us directly with the all published judgments designated as EWCOP, comprising a corpus of 411 judgments as at 31st January 2019. Three cases were excluded before analysis, as although listed with the Court of Protection case identifier (EWCOP) they had in fact been heard several years before the MCA 2005 was made law.

The remaining 408 judgments were read individually to identify when structured or objective measures of psychological or brain function were referred to in any part the judgment. Any measure which assessed a feature of psychological or brain function was included, providing there were systematic elements to the procedure which produced quantitative or other standardised outputs. This therefore could include psychometric testing of “IQ” or personality, measures of mental capacity using a structured interview format or checklist, imaging or electrophysiological study of the brain, and cerebrospinal fluid or blood investigations when used to measure brain or psychological function. Where generic statements such as “neuropsychological testing” or “a brain scan” made it clear that testing had taken place, these cases have been included, even though the nature of the test was not specified.

This generated the subset of all judgments which referred to structured measurement. Data from the judgments were then extracted using a proforma (Table 1) designed to capture 3 groups of features: circumstances of the case, presentation of structured measurement and interpretation.

**Table 1:** Proforma for collection of case features.

**Feature**	**Response**
Case ID	Numerical identifier
Case citation	*free text*
Reason for hearing	- capacity in dispute - best interests determination - other
Did the judge rule on capacity, even if the hearing was called to decide best interests?	- yes - no
Type of decision if incapacity was being considered	- healthcare or medical treatment - welfare, personal care or residence property or financial affairs - contact with individuals - marriage - sexual relations - contraception - sterilisation - to litigate - to make a will
Diagnosis of mental or physical illness for P, if any stated	*free text*
Which measurement of psychological or brain function was discussed?	*free text*
Had the measurement been used (rather than simply discussed without using it)?	- yes - no
**If a measurement was used**	
Origin of evidence of measurement	- independent expert - other professional evidence - only in background described by judge
Profession of user of measurement	*free text* (e.g., doctor including specialty, psychologist)
Evidence uses measurement to comment on impairment of mind or brain, as per MCA 2005 s.2	- presence of impairment - absence of impairment - not done - not clear
Evidence uses measurement to comment on impairment of functional decision-making, as per MCA 2005 s.3	- presence of impairment - absence of impairment - not done - not clear
Judge uses measurement to comment on impairment of mind or brain, as per MCA 2005 s.2	- presence of impairment - absence of impairment - not done - not clear
Judge uses measurement to comment on impairment of functional decision-making, as per MCA 2005 s.3	- presence of impairment - absence of impairment - not done - not clear
Judge general comments on utility of the evidence	*free text*
**If measurement was not actually used but was discussed**	
What was discussed?	*free text* (e.g., judge says they would like this measurement to be used in future)

Features of interest concerning the circumstances of the cases included the decisions which P was faced with (e.g., medical treatment, residence), whether P was reported to suffer from any “impairment in, or disturbance of, the functioning of mind or brain” and the outcome of judicial capacity determinations. Diagnostic nosology was standardised to usage of the current Chapter V of the World Health Organisation international classification of diseases (11). Multiple diagnoses were recorded and a consensus view was reached by the research team where there was dispute. Reports of merely “traits” were not recorded, due to the lack of standard criteria and variability in practice when applying this term. Such instances included several of traits of autism spectrum disorder and of personality disorders.

Features collected about the presentation of the structured measurement in court included the name of the measure, the role and profession of the person who had discussed it, and what the measure had been used to demonstrate about P. It was only occasionally possible to determine whether reports written by treating clinicians had been ordered by the court or had existed as part of the routine healthcare record, so this distinction was not retained in this study. Features collected about the interpretation of the measure by the court included whether the judge made it explicit that they were using the measurement during their determination.

Multiple judgments referring to the same case were handled in the analysis as if single cases. Where P had undergone repeated structured measurement by professionals, each new discussion of a discrete episode of use by a professional was counted. A piece of evidence mentioning multiple times a single use of a test was counted singly.

Properties of these data are summarised using descriptive statistics including frequency counts. No statistical hypothesis tests were performed. Circumstances where specific approaches seemed desired by the court are described. For additional illustrative purposes, we give a brief qualitative description of a case where measurements were reported and discussed in detail by witnesses and the judge.

## Results

### Overview

The 408 written judgments contained 146 references to structured measurement of psychological or brain function, spread over 50 persons or cases. The nature of the measurements themselves fell into three broad groups: (1) standardised tests of psychological or cognitive functioning, involving P undertaking tasks and answering questions; (2) neuroimaging and electrophysiology, such as brain scans and electroencephalography (EEG); (3) behavioural measurements of levels of consciousness, involving standardised schedules of activities undertaken with a person with reduced consciousness. Additionally, there was a single use of a measure designed to assess decision-making capacity directly. No other biomarkers were reported and no standardised measurement of symptoms of mental illness was used.

120/146 (82.2%) measurements referred to “impairment of mind or brain,” with this being as part of capacity assessment in 58/146 (39.7%). Measurement referred on 25/146 (17.1%) occasions to “functional decision-making abilities.” P had undergone structured measurement on 131/146 (89.7%) occasions, whereas on the remaining 15/146 (10.3%) occasions the potential utility of measurement was being discussed.

### Circumstances of the Cases

**Figure 1:**
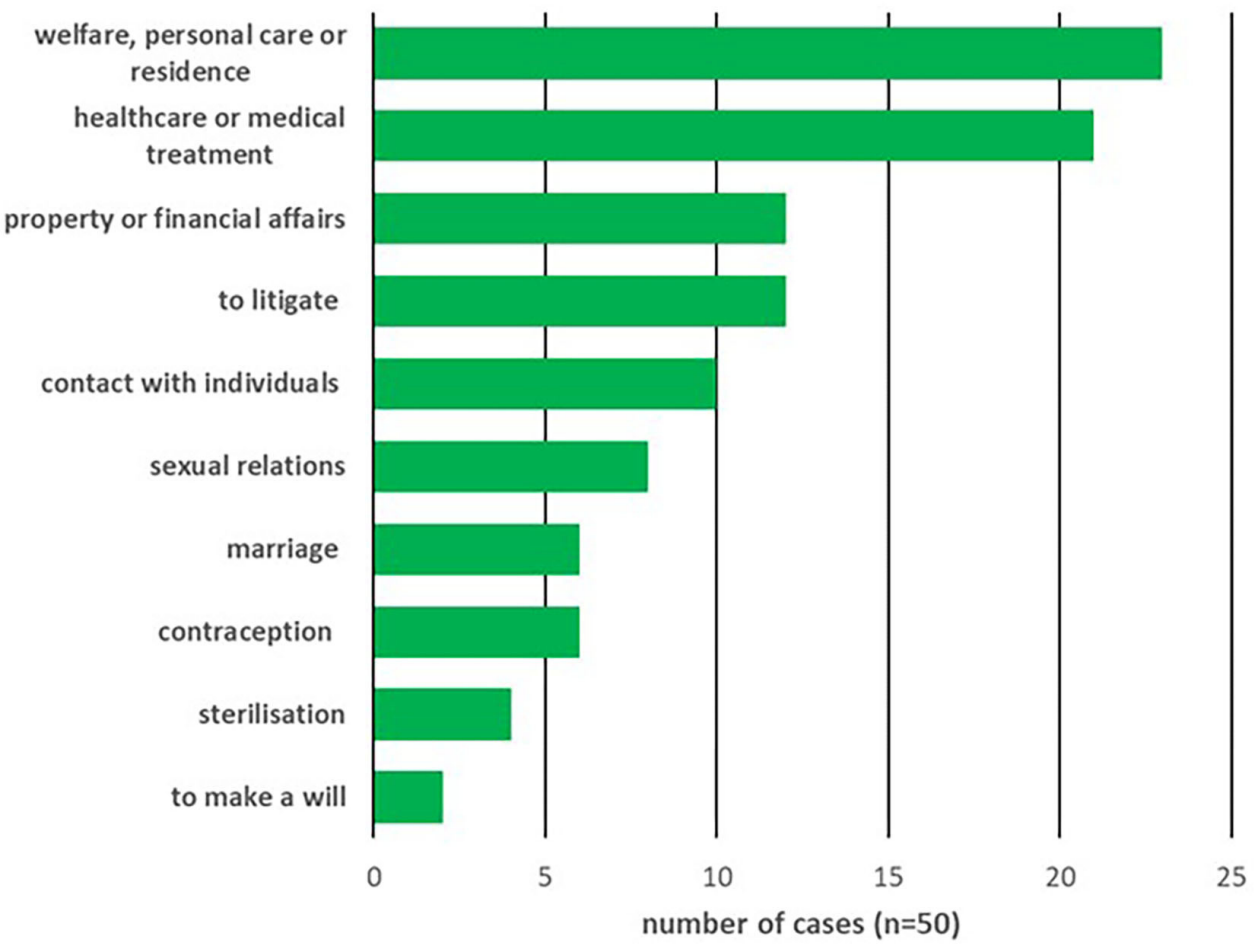
Content of decision for which capacity was in question.

Out of the 50 cases identified, in 38 (66%) the judge ruled on whether P lacked mental capacity. The content of the decisions for which these capacity determinations were being made are shown in Figure 1, with determinations about multiple categories of decision for one P being recorded multiply. Best interests were being determined in 38/50 (66%) cases using the MCA 2005 and in a further single case (2%) using the inherent jurisdiction of the court.

**Figure 2:**
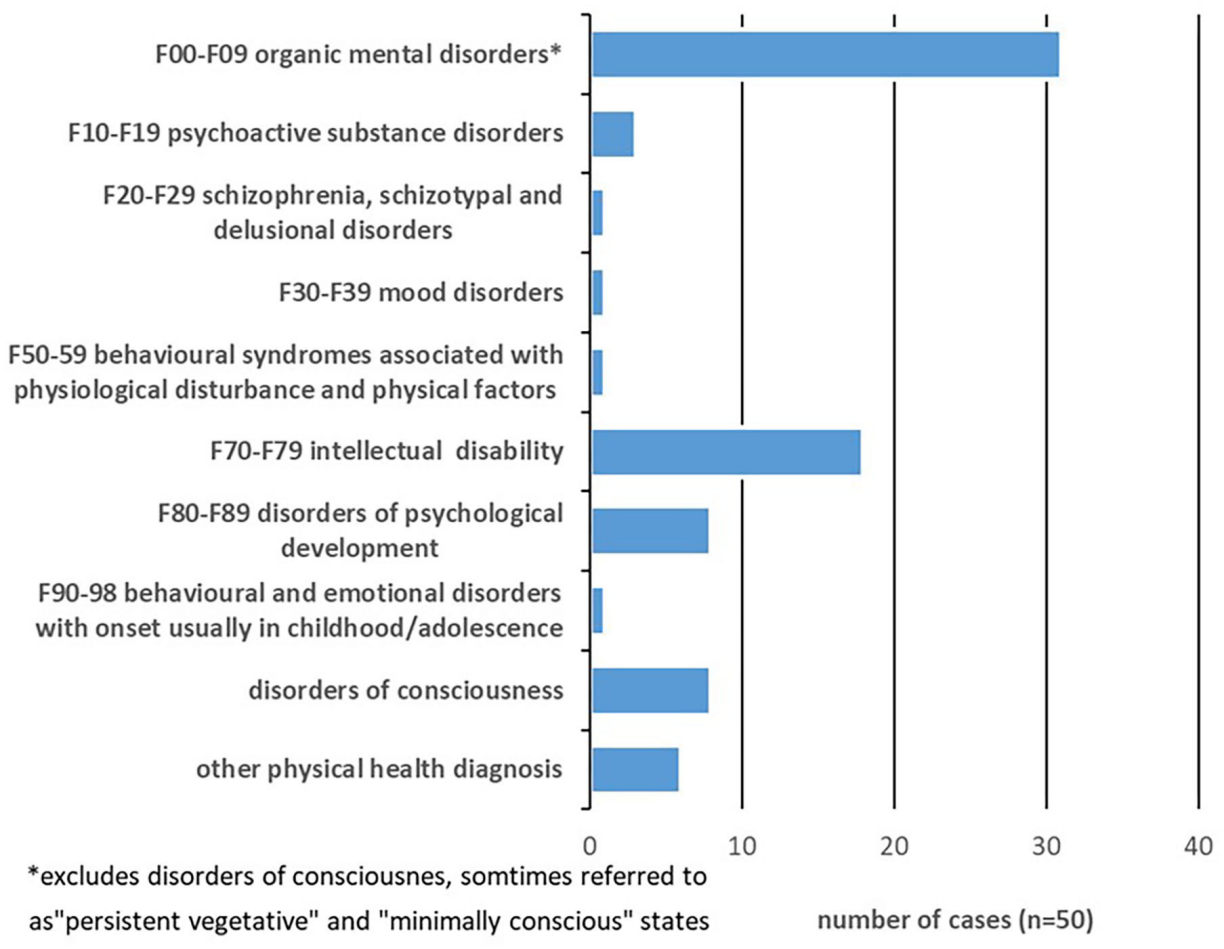
Recorded diagnoses for P.

The diagnoses reported for P in the 50 cases where structured measurement was used are shown in Figure 2, with multiple diagnoses recorded. Cases where P was labelled as suffering from a disorder of consciousness (sometimes referred to as “persistent vegetative” or “minimally conscious” states) were categorised as a separate group, because these represented a large group of cases where measurement was used for a specific and distinct purpose. For these cases, specially designed measurements were being used to determine P's level of consciousness, with the court viewing this as crucial information for determining best interests around withdrawal of treatment, nutrition or hydration (see section *Measures of Levels of Consciousness in Assessment of Best Interests* below for details of these measures). These cases comprised 12/39 (30.2%) of all cases determining best interests.

### Role in Court of Person Using Measurement

In 78/146 (53.4%) of occasions the measurement was presented by an expert witness, in 36/146 (24.7%) by a professional working with P outside of the court, in 30/146 (20.1%) by the judge without its origin being described, and in 2/146 (1.4%) by a family member of P. In no case was either P or a legal representative recorded as having referred to use of a structured measure.

In the 30 instances where the measurement was reported by the judge alone, without describing its origin, 9/30 (30%) were instances of “IQ” or intelligence quotient, 8/30 (26.7%) were of a brain scan (7 “CT” or computerised tomography, 1 “MRI” or magnetic resonance imaging), 6/30 (20%) were measures designed specifically to assess levels of consciousness, and the remaining 7/30 (23.3%) were single mentions of other measures.

### Profession of Witnesses

**Figure 3:**
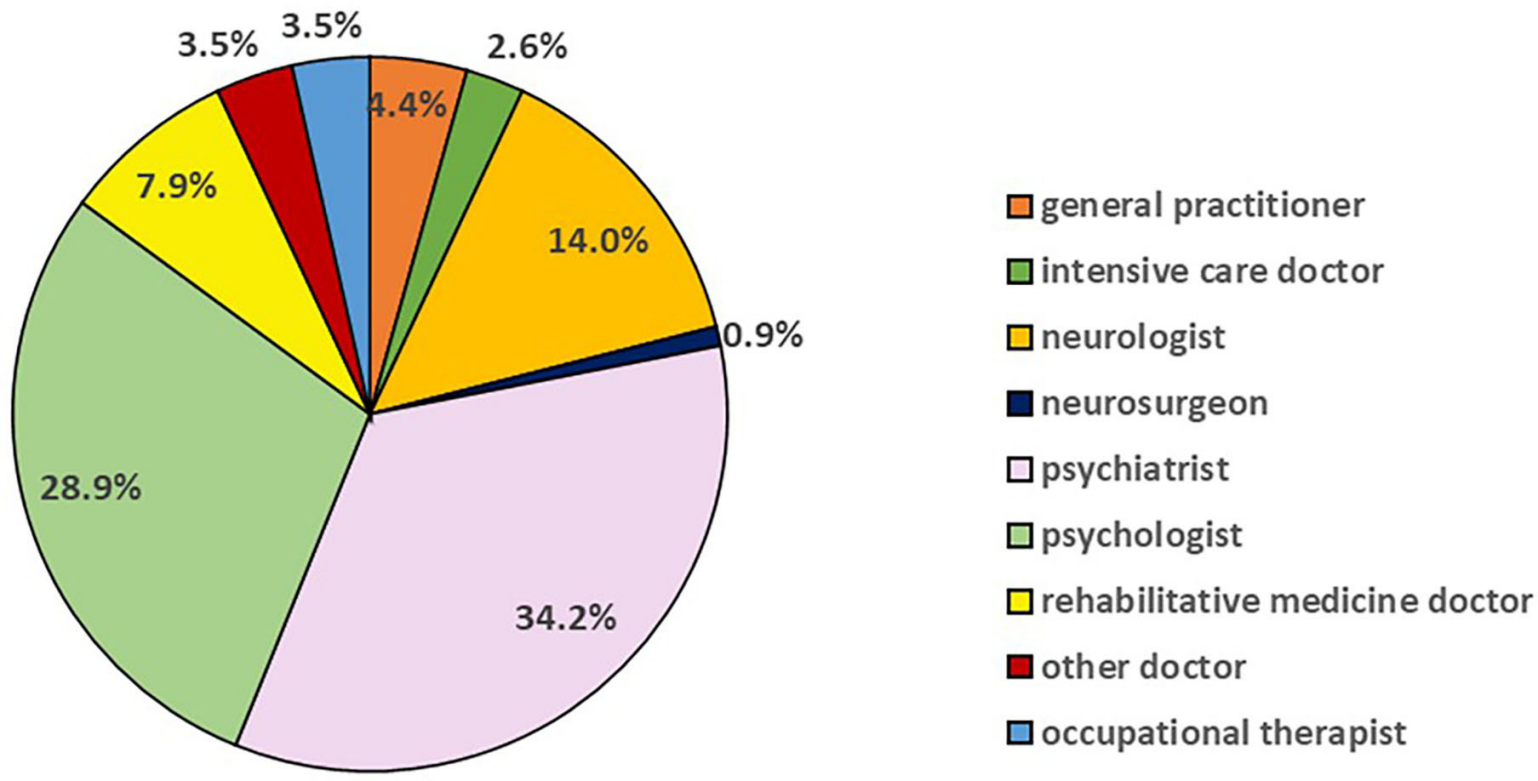
Profession of person discussing measurement.

**Figure 4:**
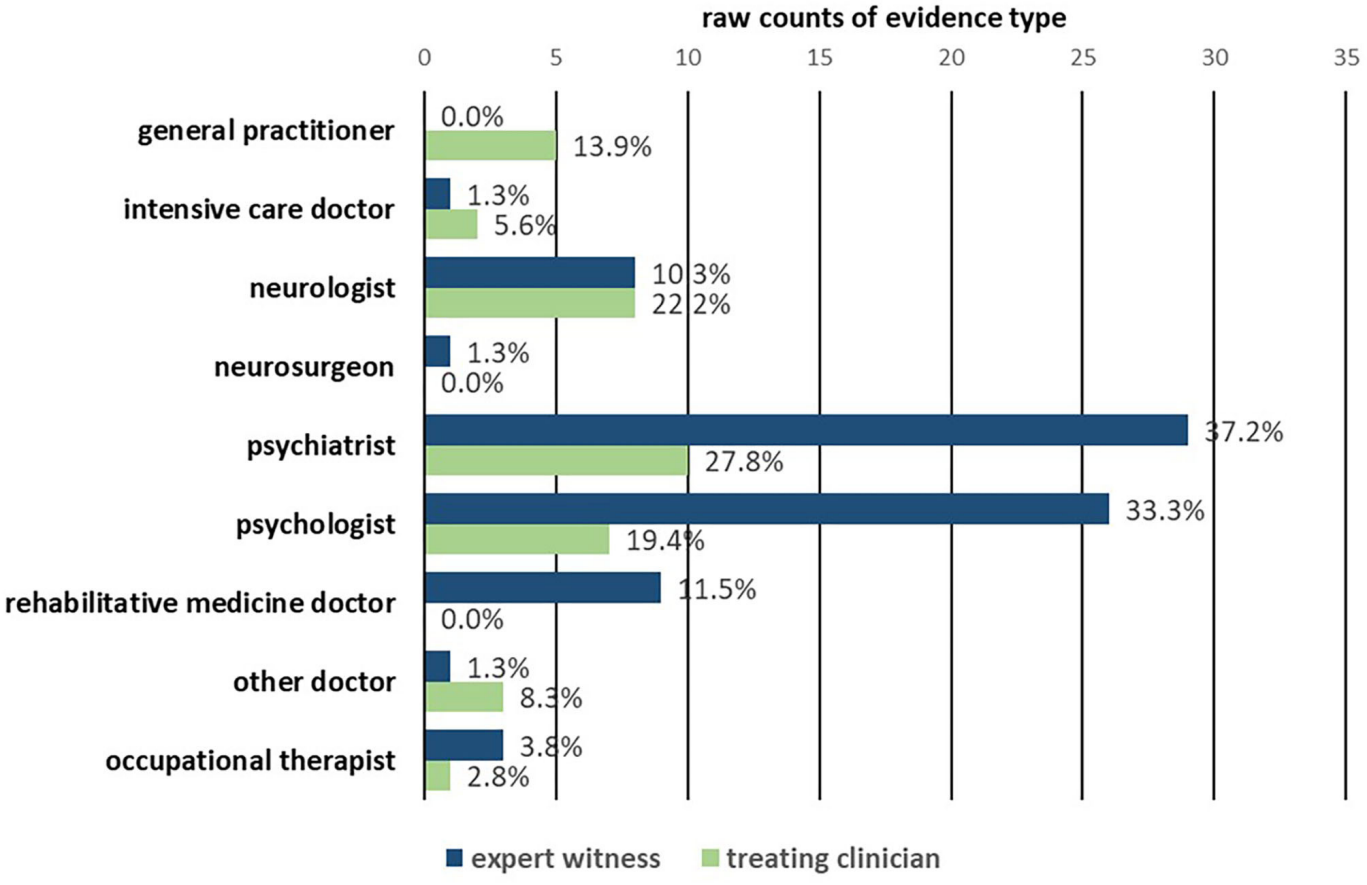
Breakdown by profession of use of measurement by expert witnesses vs. treating clinician.

The treating professionals and expert witnesses who referred to specific measurement of psychological or brain function were in every instance types of health professionals. Figure 3 depicts their professional backgrounds (*n* = 114). Structured measures were used most commonly by psychiatrists (39/114; 34.2%) and psychologists (33/114; 28.9%). Figure 4 shows whether each presentation of measurements was made by an expert witness (*n* = 78) or by a treating clinician (*n* = 36) categorised by their profession. The most frequent professional in both subgroups was psychiatrists, who contributed 29/78 (37.2%) of all structured measurement use in expert witness reports and 10/36 (27.8%) of treating clinician evidence.

### Types of Measure Used

The range of measures for testing P are shown in Table 2, with psychological measurements grouped into types, followed by measurements of levels of consciousness, and neuroimaging or electrophysiology. No other biomarkers were observed to be in use. A full itemisation of the measures used is given in Supplementary Table 1.

**Table 2:** Presentation of structured measures by an expert witness, treating professional or judge.

**Name of measure**		**Total occurrences**	**Used to assess impairment of brain or mind**		**Used to assess functional decision-making as part of a capacity assessment**	**Professional discussed reasons to use a measure without having used it in this particular case**
**Category of measure**	**Selected specific measures**		**Total**	**As part of incapacity test**		
Global cognition or intelligence		61	49	37	13	6
	*Mini-mental state examination^a^*	*15*	*12*	*11*	*4*	*1*
	*Non-specific reference to “IQ” or intelligence quota*	*23*	*17*	*12*	*3*	*1*
	*Reference to unnamed “psychometric” “neuropsychological” testing or similar*	*6*	*4*	*4*	*4*	*3*
Understanding		1	1	1	0	0
Memory		5	4	3	0	0
Frontal or executive function		8	8	8	7	1
Miscellaneous named cognitive tests or subtests		11	9	9	5	0
Capacity measure		1	0	1	1	0
Other psychological tests or too little information		4	3	2	2	1
Behavioural measures of consciousness		23	23	0	0	4
Neuroimaging and electrophysiology		21	20	0	0	3
**Grand total across all measures**		131	115	58	25	15

a *Mini-Mental State Examination (12)*.

#### Psychological Measures

Table 2 sub-categorises psychological measurements by the constructs they target. Measures with a primary focus on understanding or memory, and which appeared to have been used indeed for these purposes, have been categorised separately due to their close relationship with MCA 2005 s.3 notions of understating and retention.

Amongst the wide variety of measures used, only two were reported on more than two occasions: the intelligence quotient (IQ) measured using a variety of scales and the Mini-Mental State Examination (MMSE) (12). All other measures were discussed on a maximum of 2 occasions.

Global IQ comprised 23/131 (17.6%) of the references, either through statement of a numerical IQ value for P, or through describing the IQ test. The reference was made by psychiatrists in 7/23 (30%) occasions, psychologists in 6/23 (26%) another doctor in 1/23 (4%) and described by the judge without mention of the assessor in 9/23 (7%). There were additionally references to “verbal IQ” on 4/131 occasions (3%: 2 psychiatrists, 2 psychologists) and “performance IQ” in 2/131 (1.5%: 1 psychiatrist, 1 psychologist). These references to IQ were spread over 18 distinct individual cases. P had a diagnosis of F70-79 intellectual disability in 17/18 (94%) cases, F80-F89 disorders of psychological development in 10/18 (56%), F00-F09 organic mental disorders (disorders of consciousness) in 4/18 (22%), F30-F39 mood disorders in 1/18 (6%), F90-98 behavioural and emotional disorders with onset usually occurring in childhood and adolescence in 1/18 (6%) and an additional physical health diagnosis was named in 2/18 (11%).

The MMSE comprised 15/131 (11.4%) of references, with 10/131 (7.6%) being made by psychiatrists. Every P had a diagnosis of F00-F09 organic mental disorders, with these being, more specifically, either a dementia or an acquired brain injury in every case. In addition to quoting the total score achieved, performance on individual items or sub-sections of the MMSE was also occasionally reported.

##### Presentation of Psychological Measurement in Assessment of Mental Capacity

IQ and the MMSE were the two measures used most commonly to demonstrate both the MCA 2005 s.2 (impairment of mind or brain) and s.3 (functional inability) criteria when assessing capacity (see Supplementary Table 1). No other named measure was discussed on more than two occasions. A standardised measure of mental capacity itself was employed in a single instance. This measure was not specifically named in the judgment but was referred to as the “standard test regime” used by a particular psychologist when measuring capacity (see *YLA v PM and MZ*).

On 12/23 occasions IQ was used within an assessment of capacity to argue whether P had an impairment, but IQ was used on only 3/23 occasions to argue for P's functional inability (see Table 2). On 11/15 (73%) occasions the MMSE was used to demonstrate impairment as part of a capacity test, and in 4/15 (27%) to assess functional inability (see Table 2). The measures were accorded weight in a range of ways. A numerical IQ value was often stated as a figure at the start of a discussion and not integrated further. MMSE was often treated in a similar manner, but sometimes placed more centrally in the discussion.

#### Measures of Levels of Consciousness in Assessment of Best Interests

Four types of measurement occurred (see Supplementary Table 1). The most frequent meaures were the Sensory Modality Assessment and Rehabilitation Technique (SMART) (13) and the Wessex Head Injury Matrix (WHIM) (14). On the 23 occasions where measurement had taken place, 7 (31%) of the professionals were rehabilitative medicine doctors, 6 (26%) neurologists, 2 (9%) other doctors, and 4 (17%) occupational therapists, with the remaining 4 (17%) occurrences being in the judge's background to the case.

The SMART and WHIM were used exclusively in hearings discussing determination of best interests for withdrawal of treatment, aritifical hydration or artificial nutrition. Judgments described using these to determine whether P was in a “minimally conscious” or a “persistent vegetative” state, with withdrawal if it was determined that P was suffering from a “peristent vegeative state.” The Glasgow Coma Scale (15) was also used in such cases, but additionally was used in one other case where P had an acquired brain injury.

#### Neuroimaging and Electrophysiology

Neuroimaging and electrophysiology were presented to the court both to illustrate clinical trajectories and explain diagnostic reasoning but were not used to inform the functional test (see Table 2). On the 21 occasions where measurement had taken place, 7 (33%) of the professionals were neurologists and 3 (14%) were other doctors, with the remaining 9 (43%) occurrences being in the judge's background to the case. The 3 occasions on which a professional recommended future use of neuroimaging for P were all for diagnostic purposes and not linked explicitly to capacity assessment.

### Interpretation of Structured Measurement by the Court

Table 3 summarizes how the court interpreted the structured measurement presented to it, with measures categorised into the same types as Table 2. A full itemisation of the individual measures is presented in Supplementary Table 2.

**Table 3:** Interpretation of structured measurements by the judge.

**Name of measure**		**Judge used to assess impairment of brain or mind**		**Judge used to assess decision-making function**	**When the judge ruled on incapacity, they**	
**Category of measure**	**Selected specific measures**	**Total occasions**	**As part of a capacity determination**	**As part of a capacity determination**	**Ruled in line with evidence which used measurement**	**Ruled in opposition to evidence which used measurement**
Global cognition or intelligence		24	12	5	29.5	7.5
	*MMSE^a^*	*1*	*1*	*1*	*7.5*	*3.5*
	*Non-specific reference to “IQ” or intelligence quota*	*12*	*3*	*1*	*9.5*	*2.5*
	*Reference to unnamed “psychometric” “neuropsychological” testing or similar*	*2*	*2*	*2*	*4*	*0*
Understanding		0	0	0	1	0
Memory		1	1	0	3	0
Frontal or executive function		3	3	0	4	4
Miscellaneous named cognitive tests or subtests		0	0	0	4.5	1.5
Capacity measure		0	0	1	1	0
Other psychological tests or too little information		0	0	0	2	0
Behavioural measures of consciousness		13	1	0	0	0
	*SMART^b^*	*7*	*1*	*0*	*0*	*0*
	*WHIM^c^*	*4*	*0*	*0*	*0*	*0*
Neuroimaging and electrophysiology		8	0	0	0	0
**Grand total across all measures**		49	17	6	45	13

a *Mini-Mental State Examination (12)*.

b *Sensory Modality Assessment & Rehabilitation Technique (13)*.

c *Wessex Head Injury Matrix (14)*.

In order to consider whether P had an impairment of mind or brain, the judge integrated measurement evidence presented to them on 49 occasions. 28/49 (57.1%) of these were psychological measurements, with 12/49 (24.5%) being references to IQ. The judge discussed the measurement in reference to impairment of mind or brain (s.2) on 17 occasions. Psychological measurement comprised 12/17 (70%) and IQ 3/17 (18%). Tests of frontal/executive function were referenced on 3/17 (18%) occasions, if counted as a group.

On 6 occasions the judge referenced a structured measurement when determining functional inabilities, with all of these being psychological measures. However, on 4 of those occasions the specific name of that measure was not given.

Table 3 (final 2 columns) and Supplementary Table 2 also show for each measurement how often the judge ruled on capacity in line with the results of the measurement used to assess capacity. The judge ruled in line with the evidence in 45/58 (77.6%) of occasions, ruling in line with the MMSE on 68.2% and with use of IQ on 79.2% of occasions.

#### Brief Summary of a Case Where Structured Psychological Measurement Was Important

*D v R (the Deputy of S) and S* is an example of a case where the evidence contained much use of structured psychological measurement, and the judge discussed this use directly. This use of measurement was unusual, as the judge determined whether an inability in functional decision-making was present and both the judge and the experts discussed extensively the use of psychological testing. It was one of only a handful of such cases and was arguably the single case where functional decision-making was thus considered in the greatest level of detail.

The case concerned a person with vascular dementia of moderate severity and the judge ruled on decision-making capacity concerning property and affairs and making a will, as well as capacity to litigate. The chronology of the case was tortuous and extended, but a key decision faced by P had been the gifting of over £500,00 to a legal secretary. P had 3 times appointed enduring powers of attorney (to 3 different firms of solicitors), and the secretary, who worked for the second of these firms, had begun a friendship with P. The judge ruled separately on capacity to litigate, manage financial affairs and to make a will—and there seems to have been some use of the common law test for testamentary capacity in the expert assessments.

The case had 20 individual presentations of structured evidence, all of which were made by experts instructed for the case, with 4 being psychiatrists and one a psychologist, although one of the psychiatrists had also previously assessed P's capacity, when P had made a will. There were 6 separate references to use of the MMSE, which was used repeatedly over time, as well as targeted neuropsychological subtests. The experts chose to use different measurements, with the psychologist employing the most extensive battery of measurements, tying the outcome of the testing into the capacity assessments. He used the results of the Wechsler Adult intelligence Scale-III (WAIS-III) (16) to argue that P could not integrate new knowledge to make decisions, the semantic fluency subtest of the Repeatable Battery Assessment of Neuropsychological Status [RBAN] (17) to demonstrate “difficulties of reasoning and response control” and the Cognitive Estimates Test (18) to argue that P's “everyday verbal reasoning was grossly abnormal.”

There was disagreement between some of the experts. The experts and the judge discussed whether P's performance on components of measures was affected by other factors, such that the results would need to be re-interpreted in light of these factors—for example, that P was fatigued or that P habitually given stereotyped concrete answers because of their illness. The judge discussed at most length the use of measurements to determine the capacity to make the financial gifts. He indicated that the evidence of the psychologist was most compelling, determining on capacity in line with that evidence. The experts and judge discussed the different approaches and skills of the psychologist and a psychiatrist. The psychiatrist said that he had less training and experience in neuropsychological testing than the psychologist, and that he sometimes used measurements flexibly, deviating from their standardised modes of operationalisation. Whilst this adaptive use might have some benefits, it risked introducing variability.

## Discussion

This quantitative review of published judgments shows the different types of structured measurement of psychological and brain function presented to, and used by, the Court of Protection—a specialised court dealing with mental capacity and best interests. We have found that in only 12.5% of judgments is there reference to structured measurement of psychological or brain function. The court therefore is primarily using interpretative methods to make the determinations of mental capacity and best interests based on historical evidence, collateral evidence from informants and interviews with P.

Measurements were predominantly presented by psychiatrists (34.2%) and psychologists (28.9%). 66.4% of instances where structured measurements were presented were instances of psychological measurement, with the bulk of named measurements being of IQ and the MMSE. Measurement was often used to discuss presence of a particular impairment or diagnosis, only rarely to assess functional abilities, and never to demonstrate the causal link between these (the “causative nexus”: see *PC and NC v City of York Council*). Psychological measurement was sometimes used to assess understanding and retention, but assessment of using-and-weighing was not apparent except in isolated cases. Neuroimaging and electrophysiology were not used alone for the MCA 2005 s.3 test, which is appropriate given the psychological, rather than the anatomical or physiological, nature of the functional abilities.

In a court specialising in “impairments of mind and brain” and decision-making function, it is striking that measures of mind and brain functioning are being used so infrequently and also that judges did not often describe deliberation on or integration of measurements which could be relevant to their determinations (although it is possible that such measures are used as a matter of course in uncontentious cases, for example, to demonstrate the extent of brain damage following an injury). A notable exception is the use of standardised measurement of levels of consciousness. Best interests decisions on withholding life-sustaining treatment has been much discussed in judgments and legal journals, some of which argue that although the measures cannot be regarded as sufficient in themselves, they seem to be approaching the status of being viewed by the court as necessary evidence when considering these important decisions^2^ (20). This interest could suggest the court has an appetite for standardisation of processes in other types of case, but some features of disorders of consciousness may possibly be more amenable to measurement than mental incapacity, since they are based on observations of behaviour rather than assessments of thought processes intersecting with the will and personal preferences. Additionally, the interest of the courts in these structured measurements of level of consciousness may be partly driven by a wish to help manage the very difficult emotions which may be experienced by people involved in these important cases.

It is therefore perhaps reassuring that neuroimaging evidence is not currently used in the CoP for assessment of functional inability. Neuroimaging may be useful diagnostically and the burgeoning field of “neurolaw” reveals a global advance of its use in other types of court. For example, in criminal courts, sentences have been mitigated on presentation of neuro-evidence (21), when characteristics of a brain have been used to explain behaviour and shift responsibility. According to a neural systems view of decision-making, all decisions, both free or otherwise, are the products of brain function (22). However, several neuroethicists have raised alarm that using evidence from neuroscience to adjudicate on autonomy is non-sensical or even dangerous. Disability activists have concerns about systems which reinforce hierarchies of perceived normality, marginalising and punishing already disadvantaged groups through employing an able-bodied or able-minded perspective, which consequently focuses on the deficits it sees (23). The law may anyway require answers to questions which science is not studying (22).

Nevertheless, it has been argued that neuroimaging evidence should be used increasingly to add legitimacy to determinations of capacity in the courtroom, and neuroimaging findings have indeed been associated with impaired functional decision-making [for example (24, 25)]. There is a substantial challenge here in translating insights from laboratory decision-making tests (with or without accompanying brain imaging) and the typically one-off, emotionally-laden decisions faced by individuals subject to capacity assessments. The strength of correlation between currently available neural measures or brain imaging and situation-specific mental capacity is accordingly likely to be low. Some authors believe a prudent clinician may wish to seek out any tool which could enrich their assessment (26). Others draw caution to whether neuroscience offers objectivity fit for law, especially with public (and possibly courtroom) anxiety over attributions of certainty to science (27, 28) and little real technical guidance being available for judges as non-scientists (29).

It is not easy to extrapolate from this study to infer the landscape of measurement practices in non-court settings. A significant proportion of evidence heard in court originates in testing performed in routine healthcare. and reports written especially for the court usually involve professionals who work, or have worked, in the UK healthcare system, so the practices observed in their court reports may be reflect those they use in non-legal settings. Development of practical guidelines for the use of measurement in assessment of mental capacity, aligning legal and clinical practice, might reassure public and professionals that the relevant parameters are being captured. Structured tools such as the MacArthur Competence Assessment Tool (MacCAT) exist to score functional abilities in relation to decisions such as treatment (30) and score norms have been generated for different clinical populations. Some guidance for clinicians on how to use the MacCAT to assess for incapacity also exists (31). It was surprising therefore to see in the CoP cases that a structured measure of functional abilities with such a tool only featured once. In other areas of law, structured measurement of fitness to plead have been preliminarily evaluated in England and Wales, with the Law Commission suggesting this test be re-formulated to align with the MCA 2005, as a capacity to “participate effectively” (32, 33).

The G2i problem is a key challenge, as a measurement which ascribes to P only a probability of P having a particular state of mind or brain always fails a minority of Ps, again creating potential for disastrous personal consequences. If we never generate more than a “probability” of incapacity, while the court still requires a binary response, any assessor is left to interpret the result—presumably by performing another assessment for incapacity. The choice of neuroscientific targets for new measures needs to join up ecologically real decisions with legal thinking. The courtroom interest in IQ, MMSE and (to a lesser extent) measures of executive function, may mean that analysing the salient features of these measures is a useful starting point. There is evidence that scores on the MMSE—despite its intended purpose as a screening tool for dementia—can be strongly correlated to clinician assessments of capacity, as found in a study in a general medical hospital setting where dementia and delirium is common (34). However, this association was found to be weak in a study in a general psychiatric hospital where conditions such as schizophrenia and affective disorders, rather than dementia, are more frequent (35), suggesting the importance of clinical context. A measure developed to recommend that a person with a cognitive ability score falling below a designated cut-off is very unlikely to be able to make a treatment decision would be a good start, if it was integrated into a fuller mental capacity assessment that includes interpretation and awareness of the potential for false negatives or positives.

In the current study, few measurements focussed on using-and-weighing, yet clinicians often report this to be the most difficult construct to assess (10). Development of measurements focussing on this would thus be of interest to the law and clinicians. Another avenue for research is the concept of insight, including awareness of deficits and impairments. Measures of insight correlate with decision-making capacity (36), and although insight is frequently discussed in the CoP, it is not explicitly mentioned in the MCA 2005 (36, 37). If authentic decision-making requires a person to be aware of their own selves, then awareness of their own thinking processes could also be important. Sufficient metacognitive ability (“thinking about thinking”) might therefore be required to enable mental capacity (38) and simple methods for quantifying this are currently being studied.

## Limitations

The conclusions are particular to the legal system in England and Wales, being dependent on the legislation there. Future cross-jurisdictional research could elucidate translatable legal principles. The availability of judgments creates selection bias as only cases heard in the CoP were considered in this study. However, some cases dealing with the MCA 2005 are heard in other courts, particularly the Court of Appeal, and most do not reach any court. Analysis of practices of capacity assessment in legal settings do not therefore translate directly into health and social care settings, although these services may wish to consider the benefits of aligning their practices to court practices. The scope of our analysis was determined by the types of information and the detail in which it was described in the judgments. This meant that we could not comment on some interesting and important issues (such as the weight accorded to particular measurements by the judge) because they were described insufficiently in the data.

## Conclusions

Structured testing of brain and psychological function has been used in limited ways in the Court of Protection.

The variety of approaches used in expert witness reports demonstrates that measurement take place in non-standardised ways, risking poor reliability and validity and hence jeopardising the quality of legal decision-making. The drivers of this low level of standardisation may be complex, involving the effectiveness and availability of measurement to the relevant professions in their clinical roles and the understanding of the evidence by the court. Aligning practices of assessment in healthcare settings will facilitate translation to the courtroom more smoothly. Whilst being mindful of the practical and theoretical challenges in creating structured measures of capacity, the broad neuroscientific community should grapple with this task to improve objectivity in court processes.

## Cases Quoted

*YLA v PM and MZ* [2013] EWCOP 4020

*D v R (the Deputy of S) and S* [2010] EWCOP 2405

*PC and NC v City of York Council* [2013] EWCA Civ 478

## Data Availability Statement

Publicly available datasets were analyzed in this study. This data can be found here: https://www.bailii.org/ew/cases/EWCOP.

## Author Contributions

All authors were involved in development of the project conception, aims, and design. The data analysis was conducted by AM, who drafted the first manuscript. This was then re-written and edited extensively by SF, AD, and GO. All authors approved the final manuscript.

## Conflict of Interest

The authors declare that the research was conducted in the absence of any commercial or financial relationships that could be construed as a potential conflict of interest.

## Footnotes

^1^Other devolved nations of the United Kingdom have their own statute legislation dealing with mental capacity, through the *Adults with Incapacity (Scotland) Act 2000* and the *Mental Capacity (Northern Island) Act 2016*. However, those nations have not created additional specialist courts through their legislation on mental capacity.

^2^The authors note that use of the terms “persistent vegetative” and “minimally conscious” is viewed as outdated and pejorative by healthcare and patient groups (19).
