# Supplementary material for: The Use of Neuroscience and Psychological Measurement in England's Court of Protection
Source: Front Psychiatry. 2020 Dec 7;11:570709. doi: 10.3389/fpsyt.2020.570709 (PMC7750429; doi:10.3389/fpsyt.2020.570709)
Supplement: Supplementary file 1 [file Table_1.pdf]

Supplementary Table 1: Presentation of structured measures by an expert witness, treating professional or judge: detailed breakdown

**Name of measure**		**Total occurrences**	**Used to assess impairment of brain or mind**		**Used to assess functional decision-making as part of a capacity assessment**	**Professional discussed reasons to use a measure without having used it in this particular case**
Category of measure	Specific measure		Total	As part of incapacity test		
Global cognition or intelligence	Total	61	49	37	13	6
	Non-specific reference to "IQ" or intelligence quota	23	17	12	3	1
	Mini-Mental State Examination (MMSE) (12)	15	12	11	4	1
	Wechsler adult intelligence scale, any edition (WAIS) (39)	2	2	1	0	0
	Addenbrooke's Cognitive Examination, any edition (ACE) (40)	2	2	1	1	0
	Wechsler Intelligence Scale for Children-III (WISC-III) (41)	1	1	1	0	0
	British Picture Vocabulary Scale (42)	1	1	1	0	0
	Raven's Progressive Matrices (43)	1	1	0	0	0
	Reference to unnamed "neuropsychological" or "psychometric" testing or similar	6	4	4	4	3
	Unspecified testing of "verbal IQ"	4	4	3	0	0
	Unspecified testing of "performance IQ"	2	2	1	0	0
	Unspecified testing of "intelligence" or similar	2	1	0	0	1

	Unspecified testing of "cognition" or similar	2	2	2	1	0
Understanding	Total	1	1	1	0	0
	WAIS: comprehension subscale	1	1	1	0	0
Memory	Total	5	4	3	0	0
	WAIS: short term memory subscale	1	1	1	0	0
	Repeatable Battery Assessment of Neuropsychological Status (RBANS) (17): memory subscale	1	1	1	0	0
	RBANS: digit span subscale	1	1	1	0	0
	Unspecified testing of memory	2	1	0	0	0
Frontal or executive function	Total	8	8	8	7	1
	Behavioural Assessment of Dysexecutive Syndrome (44): rule-shift subtest	1	1	1	1	0
	Cognitive Estimates Test (18)	2	2	2	2	0
	RBANS: semantic fluency subtest	1	1	1	0	0
	Unspecified testing of verbal fluency	2	2	2	2	0
	Unspecified testing of executive function	2	2	2	2	1
Miscellaneous named cognitive tests or subtests	Total	11	9	9	5	0
	WAIS: arithmetic subtest	1	1	1	1	0
	WAIS: similarities subtest	1	1	1	1	0
	British Ability Scale (BAS) (45): reading subtest	1	1	1	0	0
	Unspecified test of reading age	1	1	0	0	0

	BAS: spelling subtest	1	1	1	0	0
	BAS: maths subtest	1	1	1	0	0
	Clock Drawing Test (46)	1	1	1	0	0
Capacity tool	Total	1	0	1	1	0
Other psychological tests or too little information	Total	3	2	2	2	1
	Abbey Pain Scale (47)	0	0	0	0	1
	Bristol Activities of Daily Living Scale (48)	1	1	0	0	0
Behavioural measures of consciousness	Total	23	23	0	0	4
	Sensory Modality Assessment & Rehabilitation Technique (SMART) (13)	15	15	0	0	1
	Wessex Head Injury Matrix (WHIM) (14)	4	4	0	0	2
	Glasgow Coma Scale (GCS) (15)	4	4	0	0	0
	JFK Coma Recovery Scale-Revised (49)	0	0	0	0	1
Neuroimaging and electrophysiology	Total	21	20	0	0	3
	Computerised tomography	12	11	0	0	1
	Magnetic resonance imaging	4	4	0	0	1
	Electroencephalography	5	5	0	0	0
	Reference to "brain scan", "neuroimaging" or similar	0	0	0	0	1
**GRAND TOTAL across all measures**		**131**	**115**	**58**	**25**	**15**
