# Supplementary material for: The Use of Neuroscience and Psychological Measurement in England's Court of Protection
Source: Front Psychiatry. 2020 Dec 7;11:570709. doi: 10.3389/fpsyt.2020.570709 (PMC7750429; doi:10.3389/fpsyt.2020.570709)
Supplement: Supplementary file 2 [file Table_2.pdf]

Supplementary Table 2: Presentation of structured measurements by the judge: detailed breakdown

Name of measure		Judge used to assess impairment of brain or mind		**Judge used to assess decision-making function** as part of a capacity determination	When the judge ruled on incapacity, they	
Category of measure	Specific measure	Total occasions	As part of a capacity determination		ruled in line with that evidence	ruled in opposition to that evidence
Global cognition or intelligence	Total	24	12	5	29.5	7.5
	Non-specific reference to "IQ" or intelligence quota	12	3	1	9.5	2.5
	Mini-Mental State Examination (MMSE) (12)	1	1	1	7.5	3.5
	Wechsler adult intelligence scale, any edition (WAIS) (39)	2	1	0	1	0
	Addenbrooke's Cognitive Examination, any edition (ACE) (40)	0	0	0	1	0
	Wechsler Intelligence Scale for Children-III (WISC-III) (41)	0	0	0	0.5	0.5
	British Picture Vocabulary Scale (42)	1	1	0	1	0
	Raven's Progressive Matrices (43)	1	1	1	0	0
	Reference to unnamed "neuropsychological" or "psychometric" testing or similar	2	2	2	4	0
	Unspecified testing of "verbal IQ"	2	1	0	2.5	0.5
	Unspecified testing of "performance IQ"	1	0	0	0.5	0.5
	Unspecified testing of "intelligence" or similar	1	1	0	0	0

	Unspecified testing of "cognition" or similar	1	1	0	2	0
Understanding	Total	0	0	0	1	0
	WAIS: comprehension subscale	0	0	0	1	0
Memory	Total	1	1	0	3	0
	WAIS: short term memory subscale	1	1	0	1	0
	Repeatable Battery Assessment of Neuropsychological Status (17): memory subscale	0	0	0	1	0
	RBANS: digit span subscale	0	0	0	1	0
	Unspecified testing of memory	0	0	0	0	0
Frontal or executive function	Total	3	3	0	4	4
	Behavioural Assessment of Dysexecutive Syndrome (44): rule-shift subtest	1	1	0	1	0
	Cognitive Estimates Test (18)	0	0	0	1	1
	RBANS: semantic fluency subtest	1	1	0	1	0
	Unspecified testing of verbal fluency	1	1	0	1	1
	Unspecified testing of executive function	0	0	0	0	2
Miscellaneous named cognitive tests or subtests	Total	0	0	0	4.5	1.5
	WAIS: arithmetic subtest	0	0	0	1	0
	WAIS: similarities subtest	0	0	0	1	0
	British Ability Scale (BAS) (45): reading subtest	0	0	0	0.5	0.5
	Unspecified test of reading age	0	0	0	0	0
	BAS: spelling subtest	0	0	0	0.5	0.5

	BAS: maths subtest	0	0	0	0.5	0.5
	Clock Drawing Test (46)	0	0	0	1	0
Capacity tool	Total	0	0	1	1	0
Too little information	Total	0	0	0	2	0
Behavioural measures of consciousness	Total	13	1	0	0	0
	Sensory Modality Assessment & Rehabilitation Technique (SMART) (13)	7	1	0	0	0
	Wessex Head Injury Matrix (WHIM) (14)	4	0	0	0	0
	Glasgow Coma Scale (GCS) (15)	2	0	0	0	0
	JFK Coma Recovery Scale-Revised (49)	0	0	0	0	0
Neuroimaging and electrophysiology	Total	8	0	0	0	0
	Computerised tomography	6	0	0	0	0
	Magnetic resonance imaging	1	0	0	0	0
	Electroencephalography	1	0	0	0	0
	Reference to "brain scan", "neuroimaging" or similar	0	0	0	0	0
**GRAND TOTAL across all measures**		**49**	**17**	**6**	**45**	**13**
